# From Big Scholarly Data to Solution-Oriented Knowledge Repository

**DOI:** 10.3389/fdata.2019.00038

**Published:** 2019-10-31

**Authors:** Yu Zhang, Min Wang, Morteza Saberi, Elizabeth Chang

**Affiliations:** ^1^School of Business, University of New South Wales, Canberra, ACT, Australia; ^2^School of Engineering and Information Technology, University of New South Wales, Canberra, ACT, Australia; ^3^School of Information, Systems and Modelling, Faculty of Engineering and Information Technology, University of Technology Sydney, Sydney, NSW, Australia

**Keywords:** knowledge repository, big scholarly data, recommender system, text mining, bibliometrics

## Abstract

The volume of scientific articles grow rapidly, producing a scientific basis for understanding and identifying the research problems and the state-of-the-art solutions. Despite the considerable significance of the problem-solving information, existing scholarly recommending systems lack the ability to retrieve this information from the scientific articles for generating knowledge repositories and providing problem-solving recommendations. To address this issue, this paper proposes a novel framework to build solution-oriented knowledge repositories and provide recommendations to solve given research problems. The framework consists of three modules: a semantics based information extraction module mining research problems and solutions from massive academic papers; a knowledge assessment module based on the heterogeneous bibliometric graph and a ranking algorithm; and a knowledge repository generation module to produce solution-oriented maps with recommendations. Based on the framework, a prototype scholarly solution support system is implemented. A case study is carried out in the research field of intrusion detection, and the results demonstrate the effectiveness and efficiency of the proposed method.

## 1. Introduction

Academic publications often reflect the development of a research field and provide classic and cutting-edge solutions to research problems. These publications generate big scholarly data that has grown exponentially since the beginning of the information age. Such “knowledge explosion” (Adair and Vohra, 2003) brings valuable opportunities for researchers to have a general understanding of the current state of development of a research problem. However, in order to find possible solutions to their problems or acquire solution-related knowledge, researchers often need to delve into a large number of articles, which is especially overwhelming for inexperienced researchers or non-professional users who only have limited knowledge of the field. Although academic searching engine such as Google Scholar and Scopus facilitate the searching process, they do not support in-depth exploration of the content and cannot mine knowledge of solutions to research problems.

There have been many studies focusing on retrieving information from the big scholarly data to understand and visualize academic papers for analysis and recommendations, such as the VOSviewer (Van Eck and Waltman, 2010), AKMiner (Huang and Wan, 2013), and AceMap (Tan et al., 2016). These systems provide useful information about the paper citation relationship and academic social networks involved in the scholarly data, however, they are not designed to retrieve problem-solving knowledge from academic papers, thereby cannot recommend solutions for given research problems. Designing efficient knowledge mining and retrieving method and scheme has long been a challenge that hinders the development of solution-oriented knowledge repositories.

In this study, three observations are leveraged to build the basis of our proposals. The first is that academic papers in most cases address one or several research problems, therefore, mining scientific solutions from an adequate number of academic papers is an effective way to find the best solution for a research problem. The second is that a good solution usually exists in a good paper that tends to have a higher impact in the field, therefore, it would be reasonable to assume that a higher impact paper is more likely to provide a better solution to a specific problem. The third is that the academic papers that propose to solve a domain (or interdisciplinary) problem often establish relationships through citations and academic social networks (authors and publication venues). Therefore, these scholarly information should be considered when evaluating the impact of a paper.

Based on the above observations, we propose a novel framework to generate a Solution-oriented Knowledge Repository (SKR) that provides scientific solutions mined from academic articles to the given research problems. To this end, we first design a semantics based information extraction module for text mining from the source articles, and propose association rules for concept mining and linking which largely improve mining efficiency compared to full text parsing. Then, a know assessment module is designed based on heterogeneous bibliometric graph to rank the collected solutions according to the impact of the corresponding articles. Finally, a SKR is generated to provide solution recommendations to each given research problem. Based on the proposed SKR framework, a prototype system, named Scholarly Solution Support System (S4), is implemented. The S4 system is tested through a case study in the field of intrusion detection. The results demonstrate the effectiveness and efficiency of the proposed method.

The novelty and contributions of this study can be summarized as follows:

- The concept of Solution-oriented Knowledge Repository (SKR) is created. It contains problem-solving knowledge that is significant for quickly understanding the development state of a research problem and finding the existing solutions for it.
- The problem of ranking scientific solutions is converted into academic paper ranking, which is solved by a ranking algorithm using the weighted Heterogeneous Bibliometric Graph (HBG).
- A Scholarly Solution Support System (S4) prototype is implemented. The case study validates that the system can automatically mine solutions from massive academic papers and provide recommendations to solve given research problems effectively and efficiently.

## 2. Related Works

Many studies have been contributed on academic article searching and recommending approaches, which can be classified into six categories including stereotyping, content filtering, collaborative filtering, co-occurrence based method, graph based method and hybrid method. These methods show advantages and shortcomings. For instance, the stereotyping (Rich, 1979; Barla, 2010; Beel, 2015) consumes a considerably large amount of human labor and time. The content filtering method (Jack, 2012; Zarrinkalam and Kahani, 2013; Ricci et al., 2015) improves the degree of system automation and accuracy by analyzing the content of scientific articles, but it creates the problems of low serendipity and high overspecialization, and it cares less about the recommendation quality. The collaborative filtering (Yang et al., 2009; Ma et al., 2014; Arapakis et al., 2015) and co-occurrence based method (Mönnich and Spiering, 2008; Gipp and Beel, 2009; Zhang et al., 2016) improve the serendipity issue but they need to deal with cold-start problem and rise computing time (Sosnovsky and Dicheva, 2010). The graph based (Bethard and Jurafsky, 2010; Le and Lauw, 2017) and hybrid approach (Burke, 2002; Lao and Cohen, 2010) utilize inherent connections within the scholarly networks, which generates higher level of recommending accuracy in general, however, employing mathematical algorithms and models increases the degree of complexity.

In addition, researchers and practitioners have proposed many academic recommending systems. ArnetMiner (Tang et al., 2008) focused on mining academic social networks, including extracting researcher profiles, incorporating publication data, modeling academic networks and providing search services for the networks. VOSviewer (Van Eck and Waltman, 2010) presented large-scale graphs displaying profiles, density and collaborative relationships of bibliometric entities. Metro maps (Shahaf et al., 2012) proposed to build road-maps for academic papers based on the metrics of influence, coverage, and connectivity generated from the papers. AKMiner (Huang and Wan, 2013) extracted the academic concepts from academic articles based on Markov Logic Networks (MLN) and constructed graphs to present their relations. AceMap (Tan et al., 2016) analyzed the big scholarly data and presented the results through a “map” in which the dynamic citation network, paper clustering, academic genealogy, author and conference homepage could be displayed. Study Map (Tao et al., 2017) proposed to reveal the knowledge learning trace of a given article based on a Reference Injection based Double-Damping PageRank (RIDP). All these systems have been developed to support users in more efficient literature review and analysis, however, retrieving the problem-solving knowledge and constructing solution-oriented knowledge repositories have not yet been explored.

Knowledge and concept mining has been studied for analyzing document content. Article Content Miner (ACM) was an outstanding example that contained an article content miner designed for assessing the quality of scientific output (Nuzzolese et al., 2016). It used the hybrid methodology including several existing technologies such as NLP, Semantic Web techniques, Ontology Design practices and FRED (Gangemi et al., 2013) enabling extraction of information from PDF documents including authors names, affiliations, countries, supplementary material, sections, tables, figures, funding agencies, and EU projects. Most of the document content extraction methods focused on mining the high-level structure of scientific articles or only extracting citation and metadata, and yet none of them have contributed in collecting the knowledge-based data from the articles (Shotton, 2009; Constantin et al., 2013; Tkaczyk et al., 2015; Perez-Arriaga et al., 2016).

This study aims to automatically find the solutions to a give research problem from academic articles, generate solution-oriented knowledge repositories, and recommend the highlighted solutions for the problem based on the impact of the articles.

## 3. Methodology

### 3.1. Definitions

**Definition 1 Research Problem (*RP*)** refers to the problem or issue that a scientific article claims to address.

**Definition 2 Proposed Solution (*PS*)** denotes the technique or approach that an article proposes to solve the issue or problem.

**Definition 3 Weighted Heterogeneous Bibliometric Graph (weighted HBG)** represents the bibliometric network that integrates scholarly information, such as papers, authors and venues of publications (journals and conferences), into one heterogeneous unit that allows them to interact with each other via sub-networks. It is worth noting that the HBG is a weighted graph considering the citation relevance and authorship. For details, see section 3.4.

**Definition 4 Solution-oriented Knowledge Repositories (SKR)** denote the knowledge bases which are composed of *RP*s, *PS*s, and the relationship between them. The *PS*s are ranked based on their impact.

**Definition 5 Association Rules** define how the papers and their corresponding *RP*s and *PS*s are linked. The rules include: (a) *RP* and *PS* are associated with the paper from which they are extracted; (b) for each paper, the *RP*(s) and *PS* are extracted from the title, abstract, introduction or conclusion, and the *PS* is associated with the *RP*(s).

### 3.2. Proposed Framework

**Figure 1:**
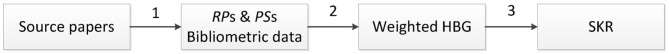
Framework illustration.

As mentioned earlier, a good solution usually exists in a good paper with a higher impact, so higher-impact papers are more likely to provide better solutions to specific problems. In other words, the problem of solution knowledge assessment can be converted into the ranking of the corresponding papers that propose these solutions. The proposed framework is illustrated in Figure 1. It takes the source articles as input. These articles are returned from Scopus by searching domain keywords defined by users. The *RP*s and *PS*s are then extracted from the papers and their corresponding bibliometric information is used to form a weighted Heterogeneous Bibliometric Graph (HBG). Afterwards, W-Rank algorithm (Zhang et al., 2019) is adopted to rank the papers, based on which the *PS*s can be assessed. Finally an SKR is generated by associating the *RP*s and corresponding *PS*s.

1. Semantics based information extraction. Run a keyword-based text mining method on the source papers to extract the *RP*s and *PS*s. In addition, the bibliometric data (citations, authors, venues, and publication time) of the corresponding papers are also extracted.
2. Weighted HBG construction. Generate a HBG by integrating the bibliometric information and employ a weighting scheme on the citation network and author-article sub-network taking into account the citation relavance and authorship to update the HBG into a weighted one.
3. Paper impact assessment (ranking) and SKR generation. Utilize a ranking algorithm, the W-Rank, to rank the corresponding papers that propose the solutions *PS*s, and finally generate a SKR by connecting the ranked *PS*s to their *RP*s based on the association rules defined at the beginning.

### 3.3. Semantics Based Information Extraction

A semantics-based text mining method using keywords is proposed in this section to extract the *PS*s and *RP*s from academic papers, where the *PS*s and *RP*s are extracted separately. Specifically, for *RP*(s), the noun terms positioned in front of the keyword are extracted since they usually denote the research problems to be addressed in an academic article. For instance, if “attack” and “intrusion” are set as keywords for searching articles in the research field of intrusion detection, we can obtain words, such as “DoS,” “DDoS,” “Flooding,” “Injection,” “eavesdropping,” and so forth using the proposed method. These words are the intrusions to be addressed in each article, which represent the *RP*s and need to be extracted. Similarly, in order to extract the *PS*s, all sentences containing the verb term “propose” or “present” or “develop” or “address” or “design” are extracted since authors commonly demonstrate their contributions, novelty or solutions by using these verbs. For instance, “*In this paper, we propose “Multilevel Thrust Filtration (MTF) mechanism” as a solution, which authenticates the incoming…* (Iyengar et al., 2014)” briefly summarizes the solution proposed in the article using the verb “propose.” The solutions or techniques proposed to solve research problems in academic articles are most likely represented in the sentences as such.

In order to reduce possible noise and improve efficiency during information extraction, only the title, abstract, introduction and conclusion of each paper are considered to the text-mining procedure rather than full text parsing. The procedure running on each paper follows a priority order, that is, the title and abstract of each paper are processed firstly, and then the introduction and conclusion. Specifically, if both *RP* and *PS* are successfully extracted from the title and abstract, the procedure stops, otherwise the introduction and conclusion will be processed until both *RP* and *PS* are found. For those papers that return partial information (including only *RP* or *PS*, or empty), they will not be considered in constructing the knowledge repository, therefore, be removed from further processing. The pseudo codes for the information extraction and association rules are shown in Algoirthm 1 which has been validated in our previous work (Zhang et al., 2018).

The extracted *PS*s and *RP*s are treated differently. When going through the text of each paper, each noun term denoting a *RP* is extracted and stored individually, resulting in one or multiple *RP*s; while the sentence(s) meeting the condition of *PS* is extracted, concatenated, and stored as one *PS*. Incorporating with the association rules, two possible scenarios could happen, including one-to-one (a pair of *PS* and *RP*) and multiple-to-one (one *PS* to multiple *RP*s). Finally, the extracted *PS*s, *RP*s and their connections will be used to develop the knowledge repository, in which the clusters in the repository are defined by the extracted *RP*s.

**Algorithm 1:**
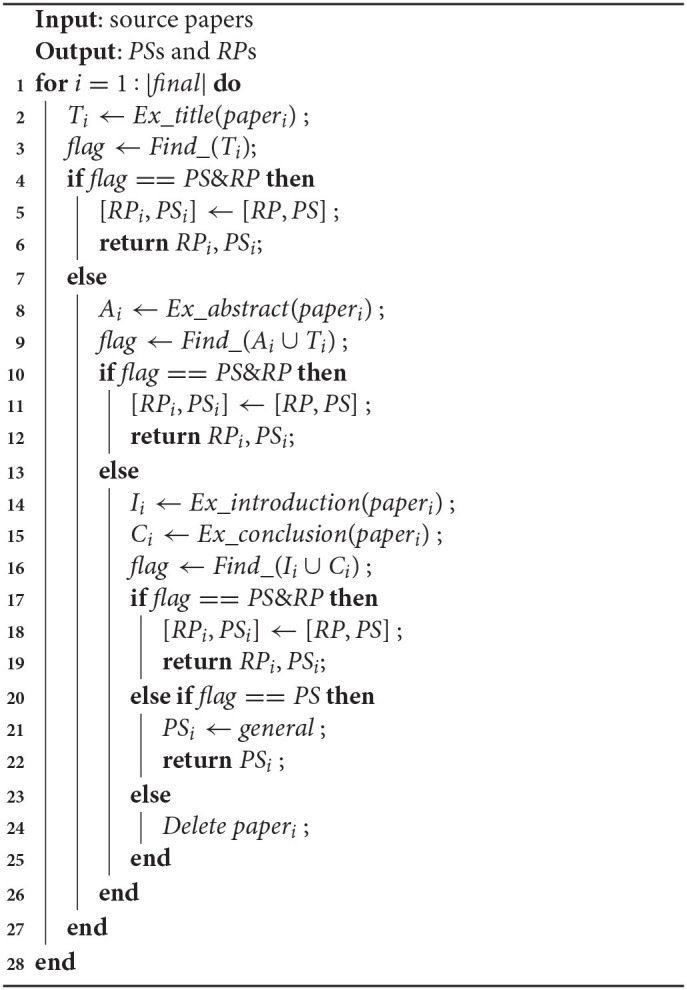
Semantics based Information Extraction

### 3.4. Weighted Heterogeneous Bibliometric Graph Construction

Recall that academic papers are not independent as they are linked to each other through citations and the academic social networks, thereby these factors should be considered when formulating an assessment of the paper impact. To achieve this, a weighted HBG is constructed using information extracted from the previous component, including the academic articles, authors, venues (journals and conferences), and the relationship amongst them.

**Figure 2:**
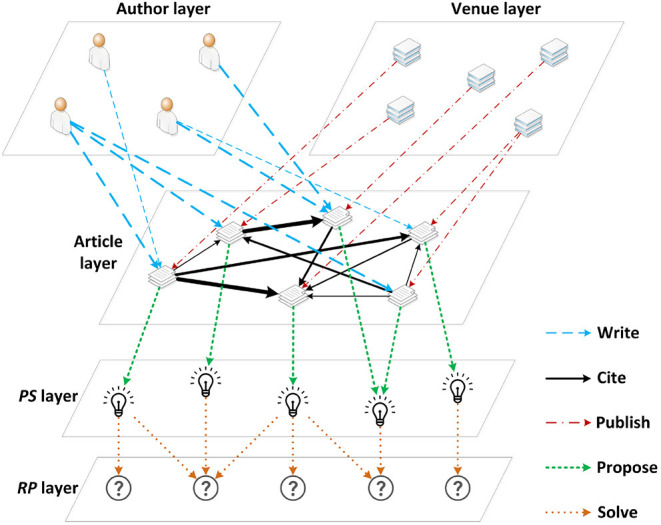
Weighted heterogeneous bibliometric graph (Weighted HBG).

The weighted HBG G is the basis of the following paper ranking algorithm and it, as illustrated in Figure 2, can be described with a set of nodes N and a set of links L connecting these nodes, as follows:

(1) $$\begin{array}{l} G=G_{P-A}\cup G_{P-P}\cup G_{P-V} \end{array}$$

(2) $$\begin{array}{l} \;=\left\{N,L\right\}=\left\{N_{A}\cup N_{P}\cup N_{V},L_{P-A}\cup L_{P-P}\cup L_{P-V}\right\} \end{array}$$

where *P*, *A*, and *V* denote article, author, and venue, respectively. Considering the citation relevance, the citation network is further updated to $G_{P-P}=\left\{N_{P},L_{P-P},\text{W}\right\}$, where **W** ∈ ℝ^*N*×*N*^ is the adjacency matrix of the citation network and $N=|N_{P}|$ is the number of articles in it. The adjacency matrix **W** is a representative description of the citation network structure with its entries, denoted as *w*_*i,j*_ referring to the relevance of a citation link from article *i* to article *j*.

The citation relevance can be interpreted from two perspectives, including the semantic similarity of the articles' content and the network-level similarity evaluating the mutual links in the citation network. For semantic similarity, we extract titles and abstracts from papers as the lexical items, and use the “align, disambiguate and walk” (ADW) algorithm (Pilehvar et al., 2013) for calculation. Titles and abstracts are selected as they contain the key information of an article, and the sense-level ADW is adopted due to its flexibility in handling lexical items in different sizes and the effectiveness in comparing the meaning of the lexical items. To measure the network-level similarity, we use Cosine similarity (Salton, 1970) as it is effective in handling citation networks. The Cosine similarity between two papers *P*_*i*_ and *P*_*j*_ is defined as follow:

(3) $$\begin{array}{l} Cosine\left(P_{i},P_{j}\right)=\frac{\left|L_{P_{i}}\cap L_{P_{j}}\right|}{\sqrt{\left|L_{P_{i}}|\times |L_{P_{j}}\right|}} \end{array}$$

where *L*_*P*_ denotes the links that connect to node *P* in the citation network, and *L*_*P*_*i*__ ∩ *L*_*P*_*j*__ the links connecting to both *P*_*i*_ and *P*_*j*_ regardless of the link direction. Finally, the citation relevance is formulated as an integration of the semantic similarity and network-level similarity according to the following equation (Zhang et al., 2019).

(4) $$\begin{array}{l} w_{i,j}=\alpha \cdot Semantic\left(P_{i},P_{j}\right)+\beta \cdot Cosine\left(P_{i},P_{j}\right) \end{array}$$

where α and β are coefficients defined by exponential functions: $\alpha =e^{\lambda \left(Semantic\left(P_{i},P_{j}\right)-\tau_{1}\right)}$ and $\beta =e^{\lambda \left(Cosine\left(P_{i},P_{j}\right)-\tau_{2}\right)}$. λ is set to 6 in favor of the similarity values which are greater than the threshold, and the thresholds τ_1_ and τ_2_ are adjusted to be the median values of the two types of similarities, respectively. The α and β are normalized so that α + β = 1.

### 3.5. Paper Impact Assessment (Ranking)

Paper ranking applies the W-Rank algorithm proposed in our previous study (Zhang et al., 2019) which outputs a list of paper scores obtained by propagating between paper authority scores *S* and hub scores *H* from three types of nodes (paper *P*, author *A*, and venue *V*) in the weighted HBG generated from the previous component. We can calculate the hub score of author *A*_*i*_ and venue *V*_*i*_ as follows:

(5) $$\begin{array}{ll} H\left(A_{i}\right) & =\frac{\sum_{P_{j}\in Out\left(A_{i}\right)}S\left(P_{j}\right)}{\left|Out\left(A_{i}\right)\right|} \end{array}$$

(6) $$\begin{array}{ll} H\left(V_{i}\right) & =\frac{\sum_{P_{j}\in Out\left(V_{i}\right)}S\left(P_{j}\right)}{\left|Out\left(V_{i}\right)\right|} \end{array}$$

where *Out*(*X*_*i*_) represents the paper nodes linked from node *X*_*i*_ in the network. Considering the citation relevance *w*, the hub score of paper *P*_*i*_ can be calculated as follows:

(7) $$\begin{array}{l} H\left(P_{i}\right)=\frac{\sum_{P_{j}\in Out\left(P_{i}\right)}w_{i,j}S\left(P_{j}\right)}{\sum_{P_{j}\in Out\left(P_{i}\right)}w_{i,j}} \end{array}$$

Based on the hub scores, we can calculate the corresponding components of authority score, namely *Citation*(*P*_*i*_), *Author*(*P*_*i*_), and *Venue*(*P*_*i*_), as follows, which are propagated from the hub scores of paper, author, and venue, respectively.

(8) $$\begin{array}{l} Author\left(P_{i}\right)=Z^{-1}\left(A\right)\underset{A_{j}\in In\left(P_{i}\right)}{\sum }H\left(A_{j}\right) \end{array}$$

(9) $$\begin{array}{l} Venue\left(P_{i}\right)=Z^{-1}\left(V\right)\underset{V_{j}\in In\left(P_{i}\right)}{\sum }H\left(V_{j}\right) \end{array}$$

(10) $$\begin{array}{l} Citation\left(P_{i}\right)=Z^{-1}\left(P\right)\underset{P_{j}\in In\left(P_{i}\right)}{\sum }H\left(P_{j}\right)w_{i,j} \end{array}$$

where *In*(*X*_*i*_) denotes the nodes linked to node *X*_*i*_, and *Z*(·) is a normalization term. In addition, we consider publishing time using the following equation to promote the prestige of new papers because they are often underestimated by citation-based models due to inadequate citations.

(11) $$\begin{array}{l} Time\left(P_{i}\right)=Z^{-1}\left(T\right)e^{-\rho \left(T_{Current}-T_{P_{i}}\right)} \end{array}$$

where ρ = 0.62, *T*_*Current*_ is the current time of evaluation, and *Z* is a normalization term. Finally, the paper authority score *S* is updated considering the above four components which are citation, authors, venues, and time according to the following equation.

(12) $$\begin{array}{l} \begin{array}{c} S\left(P_{i}\right)=\alpha \cdot Citation\left(P_{i}\right)+\beta \cdot Author\left(P_{i}\right)+\gamma \cdot Venue\left(P_{i}\right)\\ \;+\delta \cdot Time\left(P_{i}\right)+\left(1-\alpha -\beta -\gamma -\delta \right)\cdot \frac{1}{N_{p}} \end{array} \end{array}$$

where *N*_*p*_ is the total number of papers in the collection, and the last term represents a random jump. We set the four parameters so that α + β + γ + δ + θ = 0.85, which means the probability of a random jump is 0.15. The iteration procedure is summarized in Algorithm 15.

**Algorithm 2:**
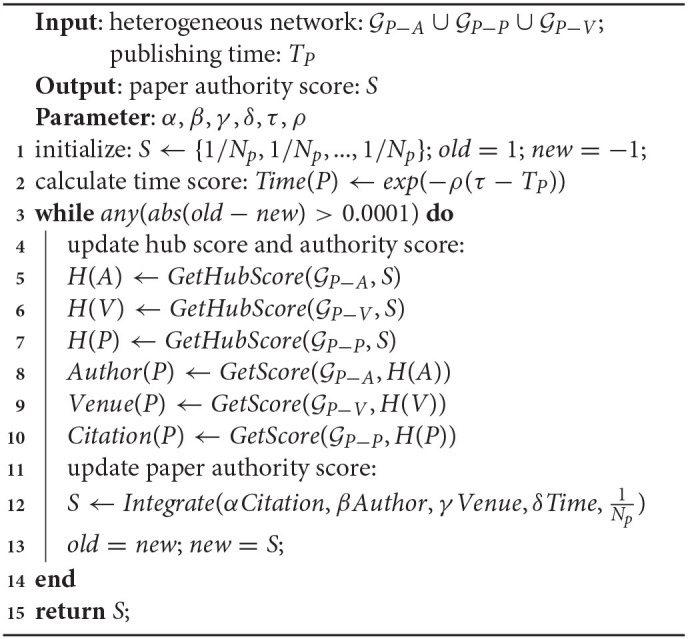
Paper Impact Assessment (Ranking)

In summary, the above paper ranking algorithm follows the four basic assumptions: (1) Papers tend to be important if other important papers cite them; (2) Authors become prestige if their articles are cited by important articles, and respected authors tend to write articles of higher quality; (3) Top venues (journals and conferences) tend to publish well-established articles, and being cited by high quality articles gives them higher impact; and (4) Articles tend to cite others for varied purposes, which produces different degrees of citation relevance. A citation is considered highly-relevant when the two papers are addressing relevant problems, using similar methods, or sharing common knowledge (Zhang et al., 2019).

### 3.6. Solution-Oriented Knowledge Repository (SKR) Generation

**Figure 3:**
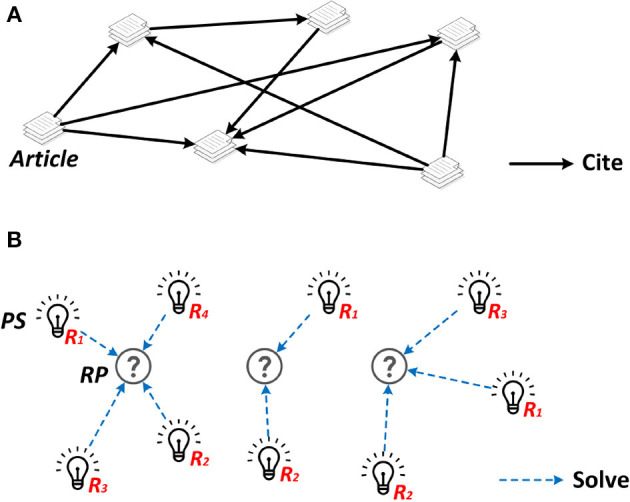
Citation network **(A)** and the proposed solution-oriented graph **(B)**.

Generation of the SKR is based on the *RP*s and *PS*s obtained by the semantics based information extraction module and the ranking results returned by the paper impact assessment module. Specifically, the *RP*s are used to generate clusters and link the corresponding *PS*s according to the association rules. Meanwhile, the *PS*s connecting to the central node *PS* in each cluster are sorted in ascending order based on the ranking result obtained from the paper impact assessment procedure. An illustration of the final SKR presented to users is shown in Figure 3B.

It is worth mentioning that a SKR is different from a bibliometric network or citation network which reflect the social relationship between bibliometric entities or the citation relationship between papers. The SKR is evolved from bibliometric network, and more importantly, it performs in-depth exploration of the paper content and mine solutions from massive data for problem-driven solution recommendation. A comparison between a citation network (bibliometric network) and our SKG is illustrated in Figure 3 in which the *R*_*n*_ refers to the ranking position of the corresponding *PS* in its own cluster. The final presentation of the SKG follows a concise design.

## 4. Case Study and Demonstration

### 4.1. Dataset and Pre-processing

The research domain of intrusion detection in cyber security was chosen to test the S4 prototype due to the fact that cyber security issues are great challenges that humans currently face and will continue to do so in the future. According to reports and studies related to cyber crimes, a great amount of economic loss has been caused by cyber security incidents and crimes, and this amount is predicted to be arising if appropriate actions are not taken (Morgan, 2018; Bissell and Ponemon, 2019). Given the massive economic loss the intrusions could lead to, the intrusion detection field is selected as the test and demonstration subject.

Scopus was utilized to collect the source papers and their bibliometric data. By applying and utilizing Scopus API key, a Python program was developed to crawl scholarly data from Scopus database. 1358 related papers were obtained in the field of intrusion detection. The bibliometric data of these papers contains 4493 authors, 1331 publication venues including journals and conferences. The citations within paper collection were obtained by collecting the citations and references of the 1358 papers, and removing those citing and referencing outside the scope of the paper collection.

In order to further process the collected papers, another program (Python) was developed which converted the PDF documents into TXT files and separated each article into section. Incorporating with the semantics-based text mining method proposed in section 3.3, the *RP*s and *PS*s denoting intrusions and detecting solutions were extracted by using the Natural Language Toolkit (NLTK) in the program.

### 4.2. Results

**Figure 4:**
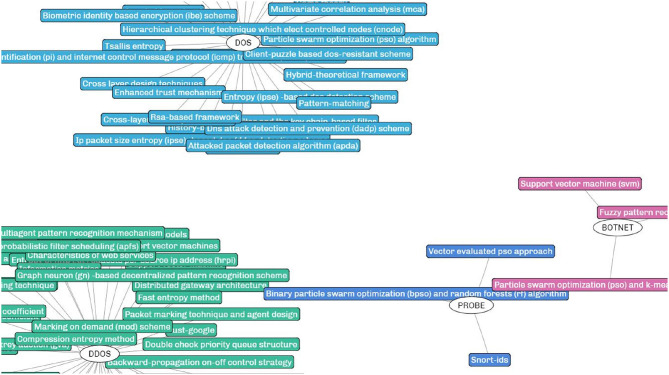
A partial view of the SKR generated for the research field of intrusion detection.

A prototype system S4 is implemented based on the proposed framework for evaluation and demonstration. A partial view of the generated SKR in the research filed of intrusion detection is shown in Figure 4. Details about the user interface and functions are specified after the result analysis.

The SKR generated by our framework is different from the existing scholarly visualizations. In the SKR, each cluster represents a research problem (the central node) with its potential solutions (surrounding nodes linked to the central node) extracted from the academic papers. For a research domain, multiple clusters can be generated depending on the number of research problems mined from the papers. In the demonstrated example of intrusion detection, the research problems *RP*s are various types of intrusions such as DOS (blue), DDOS (green), BOTNET (pink), and PROBE (indigo) to cite a few, and the surrounding squares denote the solutions (or techniques) proposed to address the corresponding intrusions.

The SKR is presented in a concise and intuitive manner, and more importantly, it rebuilds the intrinsic relationship between research problems and proposed solutions and constructs the knowledge repository for effective user recommendation. Given the significance of the solutions for problems, the repository shows great potential in both academia and industry. In addition, the implemented S4 prototype integrates several auxiliary functions such as finding the frequently discussed topics and discovering the critical research problems yet has not been fully addressed. These functions enable the system to have certain data analysis capabilities to further provide knowledge-related analytical results.

The advance of the S4 also highlights in its efficiency and automation. Table 1 shows a comparison of time consumption between the S4 and the traditional way of knowledge learning that relies on humans searching and studying a large number of articles. In the case study, the processing time of generating the final knowledge repository for intrusion detection is roughly 12 min, and during this period a number of 1358 papers has been processed. It has to be clarified that the majority time is consumed in calculating the citation relevance using semantics which is a procedure in generating the weighted HBG for the W-Rank paper ranking algorithm. The processing time can be significantly reduced to around 1 min when classic PageRank algorithm is selected (one option provided in our system), however, the ranking precision is compromised. In addition to ranked solutions to each problem, the S4 also provide a general review of the problems and solutions in this field. However, it would be overwhelming for a human to do so in limited time.

**Table 1:** Comparison between S4 automation and manual learning.

**Processing**	**S4**	**Human**
Time	12 min	Rely on human capacities
#Articles	1358	Rely on human capacities
Results	SKR with analytic report	Rely on human capacities

Regarding the output, the S4 generated a formatted knowledge repository which allows flexible user operations such as editing, adding notes, storing and downloading. But beyond all these attributes, the major contribution of S4 is that it automatically generates solution-oriented knowledge maps retrieved from academic articles, which is a distinctive feature compared to other scholarly recommending systems.

### 4.3. The S4 Prototype Demonstration

**Figure 5:**
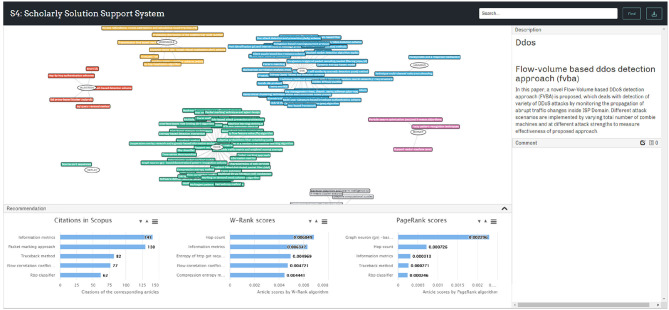
User interface of the S4 prototype.

Implementation of the prototype and User Interface (UI) design involves several programming languages, including Hypertext Markup Language (HTML), Cascading Style Sheets (CSS), and Javascript (JS), and several libraries, including JSON and Visual Notation for OWL Ontologies (VOWL). Currently the prototype is running on a local server. The system UI is shown in Figure 5. The SKR is displayed in the main panel and it is interactive. On the top right side, a node description panel is set to show the details of any selected node. A comment panel is placed at the right bottom for users to leave comments to the nodes and view the existing comments.

**Figure 6:**
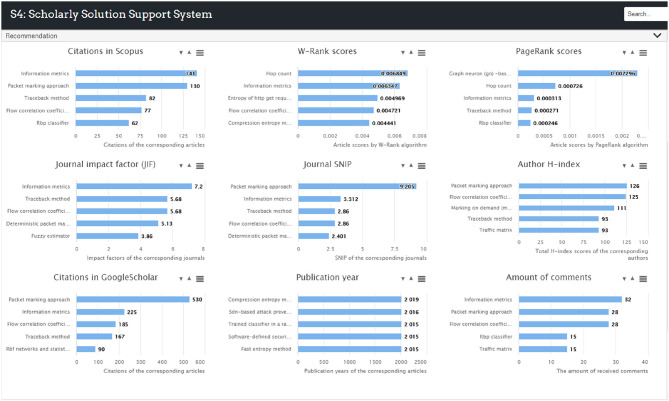
Recommendation panel of the S4 prototype.

A recommendation panel is designed at the bottom to provide analytic indexes and recommendations. This panel was developed to provide solution ranking results and recommendations to users. This function was achieved by utilizing the bibliometric information of the articles from which the *PS*s and *RP*s were extracted. Three rankings are displayed at the bottom of the S4 interface by default as shown in Figure 5 and total nine bibliometric indexes are used to rate the collected solutions as shown in Figure 6. Firstly, citation count is selected as it is by far the most widely accepted and easiest way to measure the significance of academic articles. The more times an article is cited, the more value the article is perceived to hold. Secondly, the proposed W-Rank algorithm is able to generate scores for the articles that correspond to the *PS* nodes in the knowledge map and rank them accordingly. The greater score an article obtains, the greater significant of the article. The W-Rank algorithm adopted in the system takes into account multiple bibliometric factors including citation (with citation relevance), author (with co-author contribution), publication venue, and publication time, as in Equation (12), rather than only considering paper citations. The classic PageRank algorithm is also available to rank the articles for comparison. Thirdly, the information of the corresponding journals and authors is also ranked in order to help the users make justified decisions. The article publication year and the amount of received comments are collected and made available to the users.

## 5. Conclusion and Future Work

The huge and ever growing volume of academic articles have created the “big literature,” which brings great opportunities for advancing scientific research, meanwhile it rises the difficulties for readers to find valuable problem-solving knowledge of their interests. To cope with this issue, a system that retrieves scientific solutions from academic articles and provides solution-oriented recommendations is required, yet has been overlooked in existing literature. In this study, we propose a framework to build Solution-oriented Knowledge Repositories (SKR) by semantics based information extraction and bibliometric graph based knowledge evaluation algorithms. Employing the proposed SKR framework, a Scholarly Solution Support System (S4) prototype is developed that produces a SKR in a concise, meaningful and intuitive manner and recommends scientific solutions based on their impact. The S4 prototype has been tested in the intrusion detection field, and the results validated the efficiency and effectiveness of S4 and demonstrated its potential value in both academia and industry. It automates the information retrieval and knowledge learning process, therefore, helps users in reducing their learning workload and time.

Future extension of this study will focus on the design of a document filtering module for source paper cleansing and denoising to improve the quality of the papers used in knowledge mining. By doing so, the irrelevant or low quality articles can be removed to generate a more precise knowledge repository, as well as reducing processing time. In addition, the current dataset for demonstration is not large enough, which is a limitation of this study. In our future work, we will expand our dataset in other research fields or mixed fields to verify the universal applicability of the proposed methods.

## Data Availability Statement

The datasets generated for this study are available on request to the corresponding author.

## Author Contributions

YZ proposed the conceptual framework and system design. MW and MS also contributed to the framework design. YZ and MW drafted the manuscript and figures. YZ and MW carried out the case study and experiments. MW developed the ranking algorithm. MS and EC provided supervision and support.

### Conflict of Interest

The authors declare that the research was conducted in the absence of any commercial or financial relationships that could be construed as a potential conflict of interest.
